# Shallow Marine High-Resolution Optical Mosaics Based on Underwater Scooter-Borne Camera

**DOI:** 10.3390/s23198028

**Published:** 2023-09-22

**Authors:** Yiyuan Liu, Xinwei Wang, Liang Sun, Jianan Chen, Jun He, Yan Zhou

**Affiliations:** 1 Optoelectronic System Laboratory, Institute of Semiconductors, CAS, Beijing 100083, China; 2 College of Materials Science and Opto-Electronics Technology, University of Chinese Academy of Sciences, Beijing 100049, China; 3 School of Electronic, Electrical and Communication Engineering, University of Chinese Academy of Sciences, Beijing 100049, China

**Keywords:** shallow marine optical mosaics, underwater scooter-borne camera, underwater image enhancement, superpixel-based optimal seam-line

## Abstract

Optical cameras equipped with an underwater scooter can perform efficient shallow marine mapping. In this paper, an underwater image stitching method is proposed for detailed large scene awareness based on a scooter-borne camera, including preprocessing, image registration and post-processing. An underwater image enhancement algorithm based on the inherent underwater optical attenuation characteristics and dark channel prior algorithm is presented to improve underwater feature matching. Furthermore, an optimal seam algorithm is utilized to generate a shape-preserving seam-line in the superpixel-restricted area. The experimental results show the effectiveness of the proposed method for different underwater environments and the ability to generate natural underwater mosaics with few artifacts or visible seams.

## 1. Introduction

Underwater optical imaging has been widely applied in seabed resource exploration [1], pipeline inspection [2], archaeology [3], marine biological research [4], search and rescue [5] and so on. Compared with acoustic imaging, optical imaging produces high-resolution images with more details. However, for a given optical imaging system, the total information of a single image is mainly limited by the image sensor CMOS or CCD pixels and its corresponding field of view (FOV). In other words, a high image spatial resolution is contradictory with a large FOV. Therefore, it is impossible for one single high-resolution image to cover a large-field-of-view scene. To solve this problem, underwater cameras are often equipped with divers or unmanned underwater vehicles (UUVs) for large scene awareness. Due to the complex topography and environment in shallow water, flexible divers are more suitable for shallow marine detection rather than UUVs. By means of compiling images with overlapped regions into a photo mosaic, an image stitching algorithm creates a natural high-resolution underwater mosaic with a large FOV, including all the details captured in the initial images or video frames.

Image stitching algorithms have reached a stage of maturity. There is an abundance of commercial tools, like Adobe Photoshop and AutoStitch [6], which distort images with a global homography matrix for image registration. While these algorithms perform well in cases with small parallax, they would produce artifacts such as ghosting and misalignment when stitching scenes with large parallax. Zaragoza et al. [7] proposed the As-Projective-As-Possible (APAP) algorithm for local alignment, which divides images into dense grids and warps with multiple homography matrices. However, the issue of perspective distortion in non-overlapping regions remains a challenge. To overcome this limitation, researchers have proposed the Shape-Preserving Half-Projective (SPHP) [8] algorithm, which combines APAP transformation warps with a similarity transformation. Moreover, the Adaptive As-Natural-As-Possible (AANAP) [9] algorithm adopts a smooth stitching field for alignment. This algorithm linearizes local homographies and gradually transitions to global similarity in non-overlapping areas, thereby providing precise alignment between images with varying degrees of perspective distortion. Moreover, the Seam-Guided Local Alignment (SEAGULL) [10] algorithm introduces the concept of local warp to the seam-driven [11] algorithm, and it uses the estimated seam to guide the process of optimizing local alignment and preserves the salient curve and line structures during warping. To improve color image stitching, in 2022, researchers proposed a quaternion rank-1 alignment (QR1A) model for high-precision color image alignment and an automatic color image stitching (ACIS-QR1A) framework [12] where the automatic strategy and iterative learning strategy have been developed to simultaneously learn the optimal seam-line and local alignment. Researchers have also introduced the 3D information to image stitching to combat parallax. They created one natural-looking mosaic from two overlapping images that captured the same 3D scene from different viewing positions. The 3D stitching method not only provides accurate alignment in the overlapping regions but also virtual naturalness in the non-overlapping region [13]. Since parallax is an unavoidable problem in most application scenarios, one cannot eliminate artifacts simply by registration. Consequently, post-processing technologies such as seam cutting and blending are crucial for image stitching. An optimal seam is an effective way to avoid visible stitching lines. Dynamic programming [14] and graph cutting [15] are popular methods for seam cutting. In addition, researchers have introduced superpixel segmentation to graph cutting [16,17], providing a natural seamless high-resolution panoramic image free of distortions or artifacts. Image blending is another effective technology combating artifacts. Laplacian pyramid blending [18], wavelet blending [19], average blending and linear blending are widely used fusion algorithms applied in recent years. Based on previous mature algorithms, researchers proposed a systematical applied mosaic method capable of processing up to 100 images [20]. Images are obtained from a rotated camera with little parallax. The algorithm utilizes optimal seam-line technology and multi-resolution wavelet fusion to generate coastline panoramas and solves problems of huge shadow and unnatural image connections.

The researches mentioned above show the effectiveness of the state-of-the-art stitching algorithms in dealing with a wide range of image stitching in atmospheric environments. Light absorption and backscattering from typical seawater components such as dissolved organic matter, plankton and inorganic particles limit the artificial lit area to a few square meters [21]. Since images captured by underwater cameras have low target-to-background contrast and signal-to-noise ratios, when traditional atmospheric algorithms are used for underwater photo mosaics, it easily generates panorama with misalignment and inhomogeneous illumination. Therefore, most underwater image stitching technologies have been developed based on atmospheric mosaic methods. Rajendran et al. [22] combined an unsupervised color correction based on the alpha-trimmed with Laplacian pyramid blending technique to overcome the impediments of the visible seam-line in underwater image mosaics. Different from imaging systems with fixed cameras in stable atmospheric conditions, a flexible detecting mode is suitable for underwater environments. The California Seafloor Mapping Program (CSMP) [23] created a comprehensive coastal and marine geological and habitat base map series for all of California’s state waters. Collecting images by towing underwater cameras for large scene detection, they applied gradient domain stitching and graph-cut methods, respectively, for the improvement of the illumination inconsistency and visible seams. Martin et al. [21] generated mosaics of the fjord of Trondheim at Stokkbergneset and the Ormen Lange gas field off Aukra of the Norwegian west coast. Equipped with underwater optical cameras via ROV, the researchers collected images and videos from a low-brightness and highly attenuated deep-sea region through lighting. The method utilized a Harris detector for image registration and decomposed the original images in the frequency domain by using band-pass filters for blending.

To summarize, different from atmosphere conditions, multi-image mosaics in underwater environments are still challenging in terms of the large scene awareness of the seabed, especially for images with low target-to-background contrast and low signal-to-noise ratios. Underwater scattering and absorption significantly reduce effective matches. Low-precision registration and cumulative distortion from blurry underwater images make it difficult to produce natural panoramic images with a large field of view. Under these circumstances, image enhancement, robust image registration and effective fusion algorithms are essential for high-quality underwater image stitching.

## 2. Proposed Method

**Figure 1:**
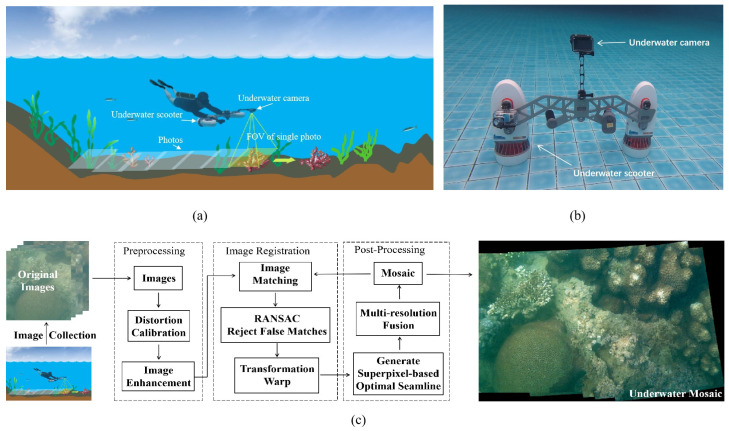
Underwater scooter-borne detection system: (**a**) image collection method; (**b**) an example of an underwater scooter-borne camera; (**c**) underwater image stitching scheme for shallow water mosaics.

The detection system is designed for the awareness of shallow marine environments, as shown in Figure 1a. Figure 1b shows an example of an underwater camera equipped with a scooter, which serves as the auxiliary device for underwater movement as well as a stabilizer to combat potential turbulence. During image collection, divers carry the facing-down underwater diver system for flexible seabed exploration. As shown in Figure 1a, images with a high spatial resolution but limited FOV are manually collected with the camera directly downward. To simplify the image stitching process, the seabed is selected as the projection plane to substitute for the final reprojection. From the images or video frames collected by the underwater camera, one can create an underwater mosaic for analysis. Based on the detection system, this paper proposes an effective image stitching method based on underwater image enhancement and a superpixel-optimized seam, especially for shallow water environments. As demonstrated in Figure 1c, the proposed method can be roughly divided into three stages: preprocessing, image registration and post-processing.

Firstly, the preprocessing involves calibration and underwater image enhancement for degraded and distorted original images. The underwater image enhancement algorithm in this paper is a variant of the classical haze removal algorithm of dark channel prior, which is based on inherent underwater attenuation characteristics. Secondly, the image registration adopts multi-precision alignment, including rough alignment for large scene awareness and precise alignment for regions containing targets, where the Scale-Invariant Feature Transform (SIFT) [24,25], K-Nearest Neighbor (KNN) and Random Sample Consensus (RANSAC) are utilized for robust feature extraction and matching. Afterwards, an appropriate transformation warp is applied for image registration. The multi-precision registration strategy is applied in our method: the similarity transformation for a global overview and the Shape-Preserving Half-Projection for high-quality alignment in the ROI. Finally, seam cutting and blending serve as post-processing for mosaic improvement. The optimal seam-line for seam cutting is obtained by dynamic programming in superpixel-restricted regions. Then, multi-resolution fusion in the neighborhood of the optimal seam is employed for final blending.

## 3. Image Preprocessing

Since underwater images traditionally have low contrast and color distortion, especially in turbid water conditions, image enhancement is necessary in the process of underwater image stitching. Dark channel prior (DCP) [26] is a classic technique used to remove haze from degraded images. As a statistical algorithm, DCP is based on the observation that some pixels have very low intensity in at least one channel of the RGB color space in most local regions that do not cover the sky. According to the observation of the dark channel, an arbitrary degraded image can be described as follows:

(1) I(x)=J(x)t(x)+A(1−t(x))

where *A* is the intensity of global light, I(x) is the observed intensity and J(x) is the scene radiance, theoretically equal to the recovered image. Moreover, t(x) represents the transmission map indicating the portion of the light that is neither scattered nor attenuated. Based on the theory of DCP, the intensity of the dark channel can be roughly interpreted as the thickness of haze. According to Equation (1), the final scene radiance can be recovered from the observed attenuated image.

**Figure 2:**
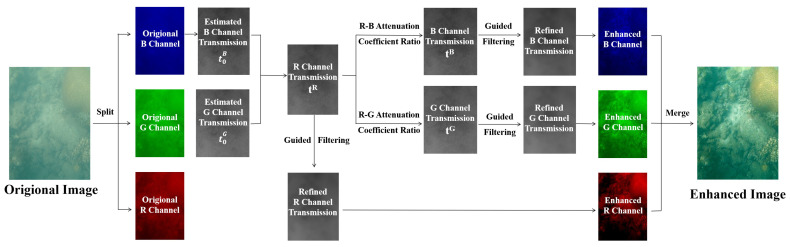
Underwater image enhancement scheme based on attenuation characteristics.

According to the Beer–Lambert law, radiance attenuates exponentially with the propagation distance of light. Underwater attenuation mainly consists of absorption and scattering. The consequence of selective degradation is that the shorter wavelengths (blue and green) propagate further than rapidly vanishing ones with longer wavelengths (red). As a result, an underwater optical image is more likely to display a characteristic bluish-greenish tone. While applying the traditional DCP algorithm, the intensity of the red channel is close to zero, providing little information on the thickness of the media. Researchers have derived the underwater dark channel prior algorithm (UDCP) [27] and red-channel underwater image restoration algorithm (RDCP) [28] that fit underwater situations, redefining a new dark channel simply considering the blue channel and green channel. To solve the problem of the selective attenuation of underwater images, this paper presents an effective underwater image enhancement algorithm based on the inherent optical properties of the water medium and the dark channel definition of UDCP and RDCP, which is shown in Figure 2.

The underwater image enhancement, shown in Figure 2, processes the attenuation of each channel. Firstly, we calculate the underwater dark channel from the less degraded blue and green channels and roughly estimate the transmission of both channels. Afterwards, we obtain the transmission of the red channel from the joint optimization of the inaccurate results of blue and green. The transmission of different channels is connected by the underwater attenuation model. Consequently, one can amend the transmission maps of blue and green light from that of the red channel. Finally, we recover the degraded image through attenuation using Equation (1).

The dark channel of the proposed image enhancement algorithm originates from blue and green light as

(2) $$J^{\mathrm{dark}}\left(x\right)=\min_{y\in \Omega (x)}\left(\min_{c\in \{G,B\}}J^{c}\left(y\right)\right),J^{\mathrm{dark}}\left(x\right)\to 0$$

where $J^{c}$ is a color channel of the scene radiance *J*, while Ω(x) is a local patch centered at *x*.

The intensity of background illumination *A* in the traditional DCP algorithm is estimated from the most haze-opaque pixels. In this paper, the components of background light are defined as the average intensity of the top 0.1% brightest pixels of each channel.

Considering underwater situations, the transmission maps of the three channels are also supposed to be estimated, and they are mathematically associated with each other. Thus, an estimated transmission map and two scalar numbers could describe the transmission characteristics of underwater images as

(3) $$\left\{\begin{array}{c} t^{R}\left(x\right)=e^{-\beta^{R}d\left(x\right)}\\ t^{G}\left(x\right)=e^{-\beta^{G}d\left(x\right)}={\left(e^{-\beta^{R}d\left(x\right)}\right)}^{\frac{c^{G}}{c^{R}}}\\ t^{B}\left(x\right)=e^{-\beta^{B}d\left(x\right)}=={\left(e^{-\beta^{R}d\left(x\right)}\right)}^{\frac{c^{B}}{c^{R}}} \end{array}\right.$$

where $\frac{c^{G}}{c^{R}}$ is the green–red attenuation coefficient ratio, and $\frac{c^{G}}{c^{R}}$ is the blue–red attenuation coefficient ratio.

The relationship between background light $A_{\lambda ,\infty }$, scattering coefficient $b_{\lambda }$ and attenuation coefficient $c_{\lambda }$ is [29]

(4) $$A_{\lambda ,\infty }\propto \frac{b_{\lambda }}{c_{\lambda }}$$

The scattering coefficient with wavelength can be approximately expressed as a linear model [30]:

(5) $$b_{\lambda }=\left(-0.00113\lambda +1.62517\right)b\left(\lambda_{r}\right)$$

where $b\left(\lambda_{r}\right)$ is a fixed wavelength for reference.

We select three standard wavelengths at 620 nm, 540 nm and 450 nm, representing the red, green and blue channels. Consequently, the attenuation coefficient ratios between different color channels can be described as

(6) $$\left\{\begin{array}{c} \frac{c^{G}}{c^{R}}=\frac{b_{G}A_{R,\infty }}{b_{R}A_{G,\infty }}=\frac{\left(-0.00113\lambda_{G}+1.62517\right)A_{R,\infty }}{\left(-0.00113\lambda_{R}+1.62517\right)A_{G,\infty }}\\ \frac{c^{B}}{c^{R}}=\frac{b_{B}A_{R,\infty }}{b_{R}A_{B,\infty }}=\frac{\left(-0.00113\lambda_{B}+1.62517\right)A_{R,\infty }}{\left(-0.00113\lambda_{R}+1.62517\right)A_{B,\infty }} \end{array}\right.$$

According to the classical DCP algorithm, one can roughly estimate the transmission maps $t_{0}^{B}$ of the blue channel and $t_{0}^{G}$ of the green channel from the dark channel in Equation (2). In this paper, the rough estimation serves as the foundation of the joint optimization of underwater transmission. Based on the attenuation coefficient ratios between different channels in Equation (6), the transmission maps $t_{0}^{B}$ and $t_{0}^{G}$ can be converted to the red channel, respectively. The mean value of the converted transmission maps is exactly the transmission map of $t_{R}$ in the red channel. Afterwards, one can amend the transmission maps $t_{B}$ and $t_{G}$ of blue and green light from that of the red channel $t_{R}$ from Equation (3). Moreover, the refined transmission is obtained from guided filtering [31]. Finally, based on the estimated transmission and background illumination, the degraded image can be recovered from the attenuation model. According to Equation (1), the image recovery is

(7) $$J\left(x\right)=\frac{I_{\max}-A}{\max\left(\tilde{t}\left(x\right),t_{0}\right)}+A$$

where $\tilde{t}$ is the estimated transmission map, and $t_{0}$ is the lower bound restricting the transmission with a typical value of 0.1.

**Figure 3:**
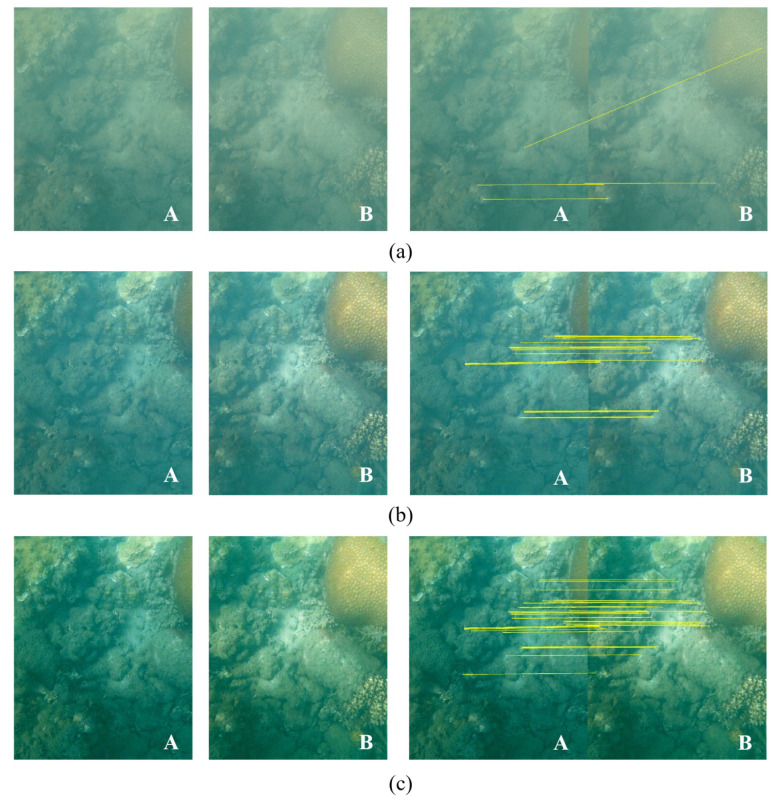
Results of underwater image enhancement and influence on matching: (**a**) original images; (**b**) DCP results; (**c**) our results.

Figure 3 shows the comparison results between the traditional DCP algorithm and our algorithm. In Figure 3, Image A and Image B are images that can be matched by their features. In Figure 3b,c, it is evident that both enhancement algorithms effectively recover the hazed details from the original images in Figure 3a. The right-hand images in Figure 3a–c illustrate the feature matching results using yellow lines. There are four matches of the original degraded images in Figure 3a. Meanwhile, after image enhancement, Figure 3b and Figure 3c display 20 and 31 matches, respectively, showing significant improvements in the image registration capability. Further analysis of the matches in Figure 3b,c indicates that there are more matches with a wider spatial distribution in our results. The matching quantity increases and the wider spatial distribution in the images is beneficial in improving the registration ability for image stitching. The comparison results show that the proposed enhancement algorithm is superior to the classical DCP algorithm in terms of the quantity and distribution of matched features in image stitching.

### 3.1. Superpixel-Based Optimal Seam-Line

Images can be roughly aligned after image registration. However, in the presence of parallax, there are problems like ghosting, artifacts and element destruction in panoramas. Therefore, researchers have introduced a dynamic programming seam cutting algorithm to address artifacts caused by inaccurate registration [14]. Kwatra et al. [15] applied the graph cuts algorithm to optimal seam-line detection. Compared with dynamic programming, the graph cuts algorithm is more complicated but also more effective in three-dimensional situations. To improve the calculation efficiency, the researchers reframed the optimal seam-line detection as a graph cuts problem in the superpixel domain instead of in the pixel domain [16,17].

To simplify the optimal seam-line algorithm, this paper combines dynamic programming with superpixel segmentation. Instead of searching for the optimal seam all over the overlapping areas, the potential optimal seam is limited to some specific regions, such as edges. Consequently, the optimal seam is a collection of split pixels that minimize the difference in the restricted fusion area. Details of the proposed superpixel-based optimal seam-line algorithm are given as follows.

1. Superpixel segmentation

The Simple Linear Iterative Clustering (SLIC) algorithm [32] is selected to produce superpixels in our method. The algorithm executes K-means clustering in the 5D space, consisting of the *r* and *l* coordinates as well as the *L*, *a* and *b* values of the CIELAB color space. The superpixel segmentation of the overlapping areas is regarded as the preprocessing for the optimal seam.

The boundaries of the superpixels form the elementary restricted region. Due to the potential for over-segmentation, morphological operation dilation is introduced to expand the scope and provide a margin for the limited optimal seam-line extension area. The typical size of the dilation filter is 5∗5 in this paper.

2. Energy definition

Based on the traditional energy definition involving texture and color, the proposed method introduces the hue difference for RGB images. The energy cost E(u,v) in our method is composed of the intensity difference $E_{int}$, the hue difference $E_{hue}$ and the gradient difference $E_{str}$ to indicate the discrepancy in the overlapped regions. The initial energy cost at pixel (u,v) is described as

(8) $$E\left(u,v\right)=\alpha E_{hue}\left(u,v\right)+\beta E_{int}^{2}\left(u,v\right)+\gamma E_{str}\left(u,v\right)$$

where α, β and γ are adjustable parameters denoting the proportions of the three factors.

Different from the traditional RGB color space, one can analyze the hue, saturation and intensity values separately in HSV. As a result, the difference in hue is calculated in the HSV color space:

(9) $$E_{HSV}\left(u,v\right)=\left|{IA}_{\;h}^{HSV}\left(u,v\right)-{IB}_{\;h}^{HSV}\left(u,v\right)\right|$$

Considering the influence of the surrounding pixels, the weighting term *F* is applied to multiply the intensity difference cost and indicates the regional intensity difference.

(10) $$E_{int}\left(u,v\right)=\frac{1}{N}\sum_{i,j\in V}\left|{IA}_{int}\left(u+i,v+j\right)-{IB}_{int}\left(u,v\right)\right|*F\left(i+1,j+1\right)$$

where *i* is the horizontal distance from the center pixel, and *j* is the vertical distance from the center pixel. In terms of the equal influence of the surroundings, the weighting term *F* is defined as

(11) $$F=\left[\begin{array}{ccc} 1 & 1 & 1\\ 1 & 8 & 1\\ 1 & 1 & 1 \end{array}\right]$$

For the gradient difference term $E_{str}$, the Sobel operator is widely used to calculate the gradient. In this paper, the Scharr operator is used as a substitute for the Sobel operator to describe the gradient difference of the overlapping areas, and one can have

(12) $$E_{str}=\sqrt{{\left(IA\left(u,v\right)*S_{U}-IB\left(u,v\right)*S_{U}\right)}^{2}+{\left(IA\left(u,v\right)*S_{V}-IB\left(u,v\right)*S_{V}\right)}^{2}}$$

(13) $$S_{U}=\left[\begin{array}{ccc} -3 & 0 & 3\\ -10 & 0 & 10\\ -3 & 0 & 3 \end{array}\right],S_{V}=\left[\begin{array}{ccc} -3 & -10 & -3\\ 0 & 0 & 0\\ 3 & 10 & 3 \end{array}\right]$$

where $S_{U}$ and $S_{V}$ represent the template of the Scharr operator.

According to Equations (8)–(13), one can initialize the energy pixels in the overlapping area.

3. Optimal seam-line research criteria

Through pixel-level energy initialization, one can transmit the difference in the overlapped regions by energy. According to the dynamic programming algorithm, the optimal seam-line is generated from the lowest cumulative energy. The specific process of the optimal seam-line method is described as follows.

Firstly, initialize the energy cost. The first row of overlapping areas is selected as the start of energy transfer, and the accumulative energy of the first row is

(14) $$E_{acc}\left(0,v\right)=E\left(0,v\right)$$

where E(0,v) is the energy cost calculated from Equation (8).

Secondly, energy transfer is a process of accumulating the energy cost from the initialized points to the last row of the overlapped domains. The cumulative energy $E_{acc}\left(u,v\right)$ of the pixel at (u,v) is related to the minimum cumulative energy of adjacent pixels, which can be described as

(15) $$E_{acc}\left(u,v\right)=\min_{k\in [v-r_{V},v+r_{v}]}E_{acc}\left(u-1,k\right)+E\left(u,v\right)$$

where E(u,v) is calculated from Equation (8), while $r_{V}$ is a parameter representing the transfer range, affecting the transmission extent.

The pixel with the lowest cumulative energy in the last row of overlapped domains is the exact solution of the optimal seam,

(16) $$tr\left(U_{cro}\right)=\underset{k\in [0,tr\left(V_{cro}\right)]}{\arg\min}\left(E_{acc}\left(U_{cro},k\right)\right)$$

where $tr(U_{cro})$ is the *v* coordinate of the optimal seam-line at the last row.

Finally, according to the solution tr(u), trace back the connected pixels to calculate the coordinates of the seam-line as

(17) $$tr\left(u\right)=\underset{k\in [tr\left(u+1\right)-r_{V},tr\left(u+1\right)+r_{V}]}{\arg\min}\left(E_{acc}\left(u+1,k\right)\right),u<U_{cro}$$

where tr(u) is the *v* coordinate of the optimal seam-line at the *u*-th row. The optimal seam-line can be recovered integrally by utilizing traversal. After this, copy the warped image to the corresponding side of the seam-line to produce a high-quality panorama.

**Figure 4:**
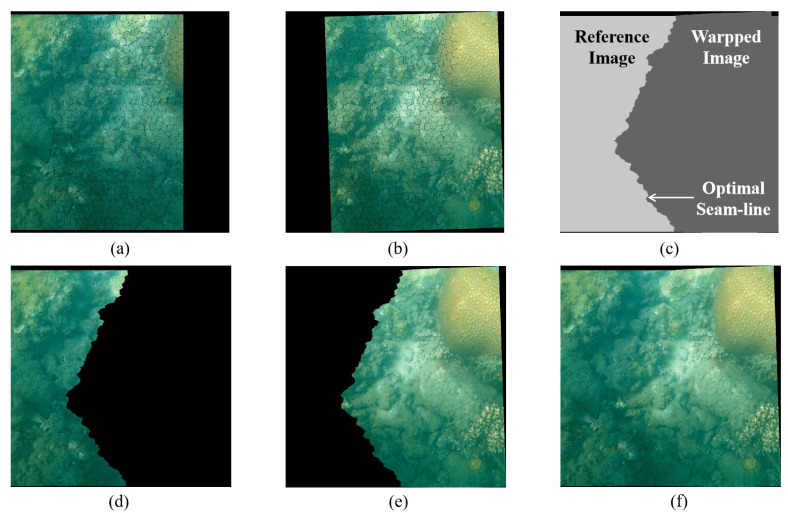
Image stitching results of different projection warps: (**a**) reference image; (**b**) warped image; (**c**) mask; (**d**) masked reference image; (**e**) masked warped image; (**f**) stitching result.

Figure 4 illustrates the implementation details of the proposed optimal seam-line algorithm. Firstly, we perform superpixel segmentation on the reference image and the wrapped image. The segmentation and dilation results are shown in Figure 4a,b. By means of traversal within the restricted regions, the optimal seam-line and a splicing mask are generated in Figure 4c from the optimal seam, dividing the plane into two parts: one from the reference image and another from the warped image. As illustrated in Figure 4d–f, according to the division principle in Figure 4c, one can copy the warped image to the corresponding side of the seam-line to generate a high-quality panorama, as in Figure 4f.

### 3.2. Multi-Resolution Fusion Constrained in Neighborhoods

Image registration and optimal seam splicing are basic steps in image stitching. However, due to misalignment in the overlapped regions, visible seams are inevitable for image mosaics. To address this issue and generate high-quality mosaics, the final step in image stitching is fusion. Mathematical models like linear fusion and average fusion may result in artifacts. Consequently, this paper adopts Laplacian pyramid fusion [18] for blending, which is performed on different scales.

For the information loss from decomposition and reconstruction, instead of conducting pyramid blending in the whole region, the area formed by 2α pixels around the optimal seam-line is defined as the region of interest (ROI) area for image fusion in our method. Based on the boundary formed by the ROI area, one can merge the fusion area with the remaining domains of the reference image and the warped image for the final panorama.

**Figure 5:**
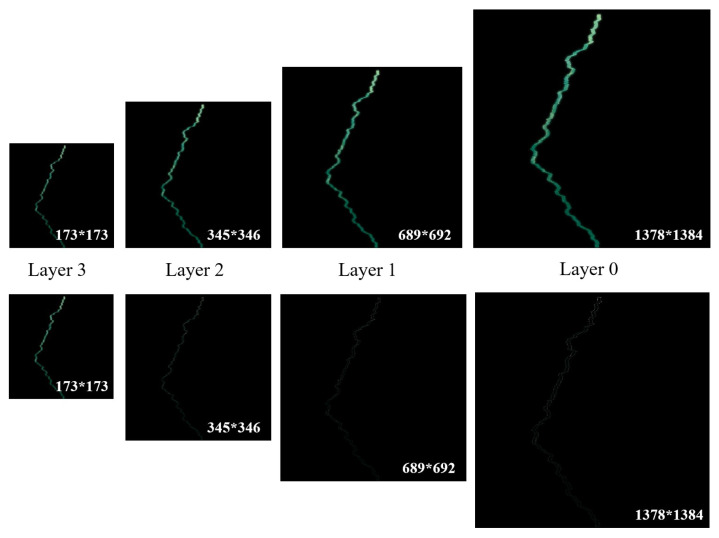
Multi-resolution fusion based on Laplacian pyramid.

In this paper, a typical value of α = 30 is used to denote the width of the ROI. As shown in Figure 5, the ROI region is a 60-pixel-wide stripe centered on the optimal seam-line. Firstly, one can build a three-layer Laplacian pyramid by down-sampling and up-sampling for the ROI region of the reference image and the warped image. Afterwards, image fusion of the original images (i.e., images of the first row in Figure 5) and the residual images (i.e., images of the second row in Figure 5) is performed from layer 3 to layer 0, respectively. Finally, the upper image of layer 0 is the result of image fusion.

**Figure 6:**
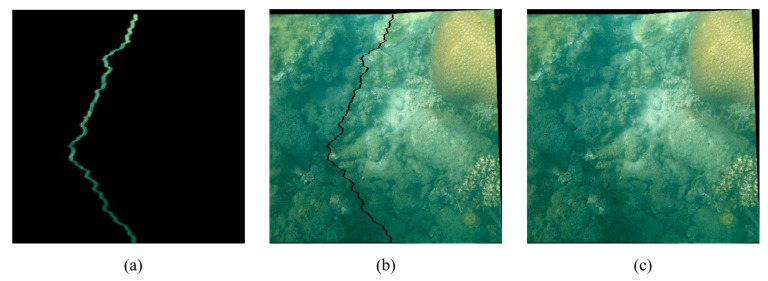
Results of image fusion and merging based on regional multi-resolution: (**a**) ROI fusion; (**b**) remaining area; (**c**) final panorama.

After Laplacian fusion in the ROI regions of Figure 6a, the proposed method selects an α-pixel image stripe centered on the optimal seam as the fusion area. The fusion area is used to replace the corresponding area in Figure 4f, and the remaining unchanged domain is shown in Figure 6b. Finally, the fusion area and the remaining area can be merged to create the final panorama, as in Figure 6c.

## 4. Experiments and Discussion

### 4.1. Experimental Environment

The experiments were all carried out in the Windows 10 operating system, with AMD Ryzen7—4800H 2.9 GHz CPU, 16GB memory environment—using Python 3.7, OpenCV 3.4.2 and numpy 1.21.2 tools. Images and videos used in the experiment were captured by a GoPro 9 and DJI OSMO Action from the Xisha Islands of the South China Sea, Hainan Province. The scooter in our experiments was the SUBLUE underwater scooter with a speed of 1.6–2.0 m/s. Since our proposed method is a universal detection technology for shallow marine environments, the camera and scooter can be replaced with other suitable commercial underwater cameras and scooters for different users.

### 4.2. Experiment Results

**Figure 7:**
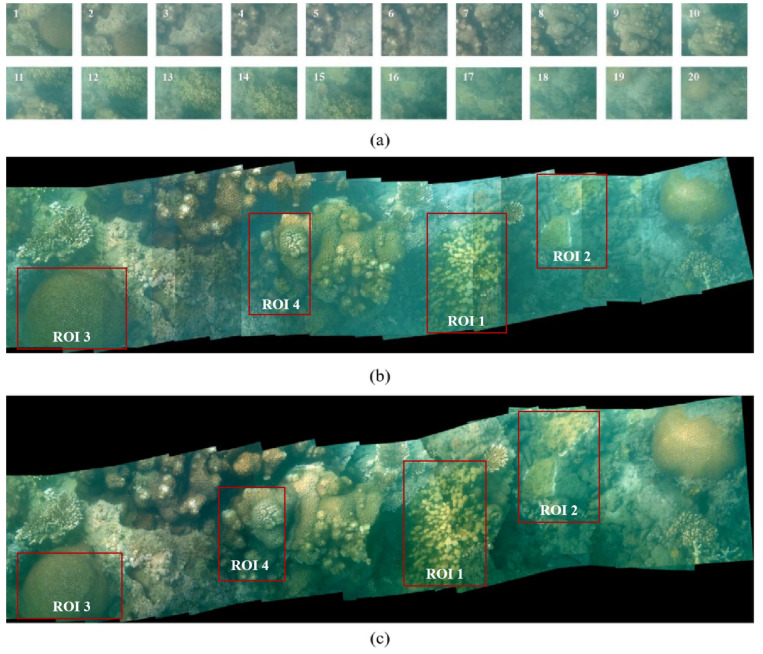
Stitching results of our proposed method: (**a**) original images; (**b**) image mosaic without fusion; (**c**) image mosaic of our method.

**Figure 8:**
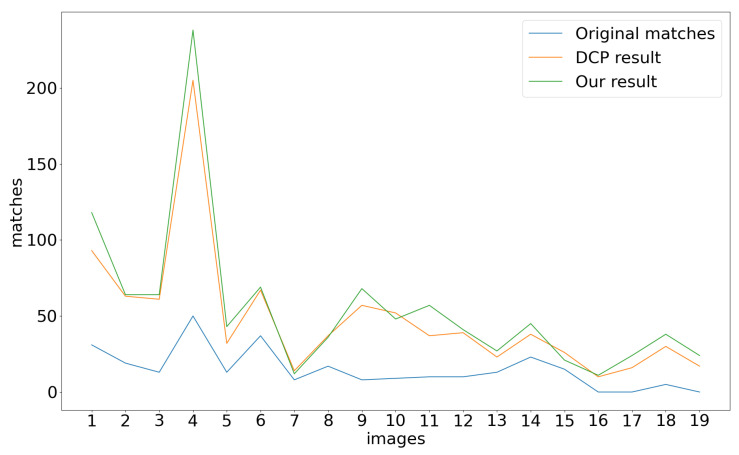
Effect of enhancement on feature matching.

The underwater image stitching method presented in this paper mainly focuses on underwater image enhancement, image registration, the optimal seam and multi-resolution fusion. As shown in Figure 7, a high-quality panorama (7037 × 2137 pixels) of Figure 7c is generated from 20 underwater images (1350 × 1080 pixels per image) in Figure 7a. In the proposed method, underwater image enhancement is vital in creating a high-quality mosaic. As specified in Figure 8, underwater image enhancement significantly increases the amount of matching features compared with the original matches without enhancement, which is of great importance for accurate registration. The blue and orange lines in Figure 8 also show that our image enhancement algorithm based on DCP and water-inherent attenuation characteristics outperforms the traditional DCP algorithm in most cases.

Effective post-processing algorithms like optimal seam-line and multi-resolution fusion are also essential for image mosaics. Figure 7b,c, respectively, show the image stitching results before and after post-processing. There are obvious ghosting and seams in Figure 7b, but they are significantly weakened by the superpixel-optimized seam and multi-resolution in Figure 7c. Table 1 shows the PSNR and mutual information quantitative comparison of four regions of interest (ROIs) in Figure 7b,c.

**Figure 9:**
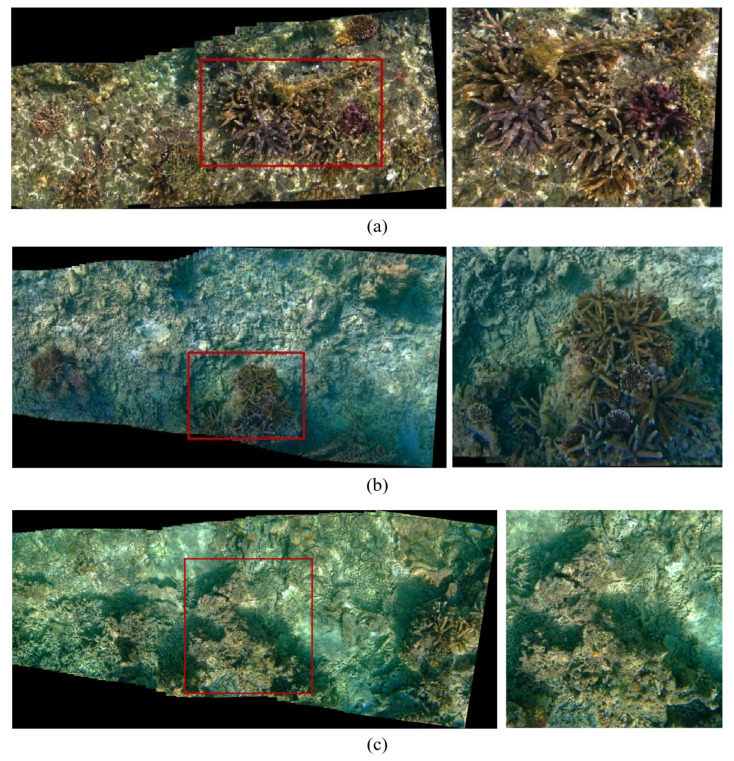
Panoramic mosaic image of seabed: (**a**) mosaic of coral area; (**b**) mosaic of block area; (**c**) mosaic of another coral area.

Figure 9 displays the stitching results of some overlapped frames with 2160 × 1080 pixels per frame from DJI OSMO Action videos. Figure 9a is a 11,877 × 5487 mosaic of an enlarged field of a coral area. Figure 9b is a 11,291 × 5873 mosaic of a block area. Figure 9c is a 14,316 × 5804 mosaic of another block area. Our multi-precision stitching strategy makes it possible to achieve large scene awareness and detail perception.

As shown in Figure 1a, images are manually collected with the camera directly downward without pose sensors, and thus the shooting angle is not strictly straight. However, the results of Figure 7 and Figure 9 show that the proposed underwater image stitching method is effective for different underwater conditions. Note that the parameters in our experiment are simply reference values. The parameters of DCP are typical values, which perform well in underwater image enhancement. The grid size and the range of the optimal seam-line are parameters related to the size of the original images, which should be tuned for different image sizes. The panoramic images provide valuable insights for seabed mapping, the convenient observation of regional corals, the statistical analysis of coral coverage and species identification.

## 5. Conclusions

This paper describes a shallow marine high-resolution optical mosaics acquisition technique using an underwater scooter-borne camera. The underwater image stitching method for seabed exploration and coral detection has been developed based on underwater image enhancement and superpixel-optimized seam algorithms. Firstly, we utilize an underwater image enhancement algorithm for higher contrast and reduced color distortion, which is based on the dark channel prior algorithm and inherent underwater attenuation characteristics. Afterwards, we search for the optimal seam over the superpixel-restricted regions of overlapping areas and perform Laplacian pyramid multi-resolution fusion for the image stripe centered on the optimal seam-line. Finally, the quantitative analysis of the feature matching and stitching results in various underwater situations illustrates the effectiveness of our method.

Instead of analyzing blurry images or videos with a limited FOV, the underwater mosaic of our method provides a convenient and distinct tool for underwater research. The technology for underwater image stitching for high-resolution and large-FOV contexts is still in its early stages, and the proposed method is useful for shallow marine exploration and research.

## Figures and Tables

**Table 1:** Quantitative analysis of image mosaics.

ROI	PSNR(b) ^1^	PSNR(c) ^2^	Mutual Information(b) ^1^	Mutual Information(c) ^2^
ROI1	14.68	15.10	0.54	0.61
ROI2	17.29	19.96	0.70	1.49
ROI3	19.22	21.73	0.83	1.49
ROI4	17.31	18.75	0.79	1.17

^1^ PSNR and mutual information of Figure 7b; ^2^ PSNR and mutual information of Figure 7c.

## Data Availability

The data are unavailable due to privacy concerns.
